# Editorial: Novel mechanisms involved in urinary bladder control: advances in neural, humoral and local factors underlying function and disease, volume III

**DOI:** 10.3389/fphys.2025.1576452

**Published:** 2025-02-27

**Authors:** Monica A. Sato, Russ Chess-Williams, Patrik Aronsson

**Affiliations:** ^1^ Department of Morphology and Physiology, Faculdade de Medicina do ABC, Centro Universitario FMABC, Santo Andre, Brazil; ^2^ Faculty of Health Sciences & Medicine, Bond University, Gold Coast, QLD, Australia; ^3^ Department of Pharmacology, Institute of Neuroscience and Physiology, University of Gothenburg, Sahlgrenska Academy, Gothenburg, Sweden

**Keywords:** urinary bladder, overactive bladder, medial preoptic area, ectonucleotidases, tibial nerves, sickle cell disease (SCD), pituitary adenylate cyclase-activating polypeptide (PACAP), nitric oxide

This Research Topic brings several novelties about physiological and pathological aspects focusing on the urinary bladder. In the third volume about this subject that follows the earlier volumes (Sato et al., 2020; Sato et al., 2022), the different authors show with multiple angles, from the molecular to the systemic level, a variety of features displayed by the urinary bladder, allowing the reader to unravel the physiology of this fascinating organ.

Regulation of the urinary bladder is classically known for being dependent on both central and peripheral mechanisms. Although it has been deemed that the neural mechanisms involved in urinary bladder control are well stablished (De Groat et al., 2015), in recent years, novel central areas and their neurotransmissions have revealed that much more needs to be uncovered regarding these central mechanisms. Daiuto et al. have shown that the medial preoptic area (mPOA) is involved in urinary bladder regulation through a phasic mechanism in female Wistar rats. In this brain area, it is the angiotensinergic transmission by activation of AT-1 receptors, but not the GABAergic neurotransmission, that mediates the intravesical pressure control.

Peripherally, the urinary bladder is innervated by the autonomic nervous system. Burnstock (2014) has shown that adenosine 5′-triphosphate (ATP) is released as a co-transmitter with acetylcholine from parasympathetic nerves and also is likely a co-transmitter with norepinephrine/noradrenaline from the sympathetic innervation of the bladder. The release of ATP by efferent neurons can modulate smooth muscle tone (Vial and Evans, 2000). In contrast, urothelial ATP can act on suburothelial interstitial cells/myofibroblasts (Wu et al., 2004; Cheng et al., 2011), in autocrine and paracrine ways to stimulate urothelial cells (Ferguson et al., 2015; Chess-Williams et al., 2019), and sensory nerves (Cockayne et al., 2000; Kaan et al., 2010). The activation of purinergic receptors on sensory nerves is thought to convey the sensation of bladder fullness and onset of the micturition reflex (Cockayne et al., 2000; Kaan et al., 2010). However, released ATP is easily hydrolyzed by membrane-bound and soluble forms of ectonucleotidases to ADP, AMP, and adenosine (ADO) (Yu, 2015; Aresta Branco et al., 2022; Gutierrez Cruz et al., 2022). Particularly, ADP and ADO are biologically active metabolites that can modulate detrusor function, in which ADP actions result in detrusor muscle contraction (Yu et al., 2014), whereas ADO causes smooth muscle relaxation (Hao et al., 2019). Branco et al. have used RNAscope™, an RNA *in situ* hybridization technology, to demonstrate the distribution and measure the levels of ectonucleotidases gene expression in large high-resolution images of murine bladder sections. They suggested that layer-specific differences of ectonucleotidases gene expression found in their study could be relevant for regulation of purine availability and subsequent functions in the bladder wall.

Interestingly, animal studies have shown that the afferent tibial nerve may be also responsible for bladder modulation (Kovacevic and Yoo, 2015). Evidence suggests that unmyelinated C-fibers, but not Aδ or Aβ-fibers, were recruited during tibial nerve stimulation in a continuous-fill rat model (Paquette and Yoo, 2019). Zhou et al. investigated if the stimulation of C-fibers in tibial nerves can induce bladder inhibition by optogenetic transdermal illumination by cystometric evaluation. They demonstrated that prolonged bladder inhibition is mediated by the stimulation of C-fibers in the tibial nerves, with no frequency-dependent characteristics, and suggested that 473-nm blue light has limited penetration efficacy, but it is enough to modulate bladder functions through transdermal illumination on the superficial peripheral nervous system.

Pathological conditions affecting the urinary bladder and the understanding of mechanisms underlying these disorders have been also focused in this Research Topic. Sickle cell disease (SCD), an autosomal recessive genetic disorder that causes abnormal hemoglobin S (HbS) production due to a single amino acid substitution in the β-globin chain, can be evoked by genetic mutation. This triggers the polymerization of HbS under hypoxic or dehydrated conditions, forming sickle-shaped erythrocytes (Kato et al., 2018). Such altered cells exhibit increased stiffness and a reduced lifespan, leading to intravascular and extravascular hemolysis, which are critical features of SCD and yielding several clinical manifestations (Kato et al., 2018). A relevant molecular consequence of intravascular hemolysis is the reduction of nitric oxide (NO) bioavailability due to direct NO-hemoglobin interactions and increased reactive oxygen species (ROS) production, which act as NO scavengers (Reiter et al., 2002; Vona et al., 2021). This reduction in NO availability is associated with severe SCD complications, including the overactive bladder (OAB) (Kato et al., 2017). Rebecchi e Silveira et al. have investigated the effects of intravascular hemolysis on the micturition process and the contractile mechanisms of the detrusor smooth muscle (DSM) in a mouse model with phenylhydrazine (PHZ)-induced hemolysis. In addition, the role of intravascular hemolysis in the dysfunction of nitric oxide (NO) signaling and oxidative stress in the bladder was evaluated. They demonstrated that intravascular hemolysis promotes voiding dysfunction that correlated with alterations in the NO signaling pathway in the bladder. In addition, increases in oxidative stress were evoked by intravascular hemolysis. They suggested that intravascular hemolysis elicited an OAB phenotype similar to those observed in patients and mice with SCD.

Another pathological aspect has been shown by Oliveira et al. in this Research Topic. As hyperglycemia in diabetic individuals causes accumulation of the highly reactive dicarbonyl compound methylglyoxal (MGO), which modulates TRPA1 activity, and long-term oral intake of MGO causes mouse bladder dysfunction, they investigated the TRPA1 expression in the bladder and the effects of 1 h-intravesical infusion of the selective TRPA1 blocker on cystometric alterations induced by MGO. Their findings demonstrated that TRPA1 activation is implicated in mouse overactive bladder induced by MGO, and suggested that TRPA1 blockers could be useful for the treatment of diabetic bladder dysfunction in individuals with high MGO levels.

Tay and Grundy bring a review about the different animal models of interstitial cystitis/bladder pain syndrome (IC/BPS), and the mechanisms underlying these models. They highlight that many of the animal models mimic the major symptoms of IC/BPS. The refining of these models to induce chronic symptomatology can resemble the IC/BPS phenotype, nevertheless, it is noteworthy that no single model can fully replicate all aspects of the human disease. Thus, likely different models still will be necessary for preclinical drug development, depending on the unique etiology of IC/BPS under investigation.

Ke et al. have demonstrated the role of Pituitary Adenylate Cyclase-Activating Polypeptide (PACAP) and its receptor PAC1 in IC/BPS and the potential involvement in inflammation and sensory dysfunction. Using different approaches such as transcriptomic analysis, immunohistochemistry, and bladder function assays, Ke et al. assessed the possible correlations between PACAP/PAC1 activation, bladder inflammation, and sensory dysfunction. In addition, the modulation of the PACAP/PAC1 pathway was evaluated in rats to determine its effects on bladder inflammation and function. The findings suggested that the PACAP/PAC1 pathway is involved in the inflammatory and sensory changes observed in IC/BPS, opening perspectives for the development of new targeted treatments.

Another study by Aronsson et al. used a method to produce NO in an aqueous solution and validated its capacity to induce functional responses in isolated rat bladders, as well as comparing the NO responses to the commonly used NO donor sodium nitroprusside (SNP). The authors also evaluated the impact of ongoing inflammation on the involvement of soluble guanylate cyclase (sGC) dependent signaling in NO relaxation. They found that aqueous NO solution induces relaxation of the rat detrusor muscle by activating sGC in both control and inflamed bladder strips in an experimental cystitis induced by a single injection of cyclophosphamide. However, inflammation possibly leads to decreased sGC expression in the detrusor muscle. The authors emphasize the usefulness of the aqueous NO solution as a valuable pharmacological tool for studies of the lower urinary tract.

In conclusion, this Research Topic covers an array of studies addressing physiological and pathological aspects of the urinary bladder. We believe that the enriching lessons of the valuable approaches shown here will raise novel issues as well as open new avenues for further studies that may ultimately lead to novel therapies for patients with bladder dysfunctions.
